# Perceived Prejudice and the Mental Health of Chinese Ethnic Minority College Students: The Chain Mediating Effect of Ethnic Identity and Hope

**DOI:** 10.3389/fpsyg.2017.01167

**Published:** 2017-07-11

**Authors:** Jin Yao, Liping Yang

**Affiliations:** School of Psychology, Nanjing Normal University Nanjing, China

**Keywords:** perceived prejudice, ethnic identity, hope, mental health, China, minority ethnic groups, college students

## Abstract

As a multinational country incorporating 56 officially recognized ethnic groups, China is concerned with the mental health of members of minority ethnic groups, with an increasing focus on supporting Chinese ethnic minority college students. Nevertheless, in daily life, members of minority ethnic groups in China often perceive prejudice, which may in turn negatively influence their mental health, with respect to relative levels of ethnic identity and hope. To examine the mediating effects of ethnic identity and hope on the relationship between perceived prejudice and the mental health of Chinese ethnic minority college students, 665 students (18–26 years old; 207 males, 458 females; the proportion of participants is 95.38%) from nine colleges in the Guangxi Zhuang autonomous region and Yunnan and Guizhou provinces of China took part in our study, each completing adapted versions of a perceived prejudice scale, a multiethnic identity measure, an adult dispositional hope scale, and a general health questionnaire. Analysis of the results reveals that perceived prejudice negatively influences mental health through both ethnic identity and hope in Chinese ethnic minority college students. The total mediation effect was 54.9%. Perceived prejudice was found to negatively predict ethnic identity and hope, suggesting that perceived prejudice brings about a negative reconstruction of ethnic identity and hope mechanisms within the study's Chinese cultural context. The relationship between perceived prejudice and mental health was fully mediated by hope and the chain of ethnic identity and hope. Ethnic identity partially mediated the relationship between perceived prejudice and hope. The relationship between perceived prejudice and mental health mediated by ethnic identity was not significant, which suggests that the rejection–identification model cannot be applied to Chinese ethnic minority college students. This paper concludes by considering the limitations of our study and discussing the implications of its results for researchers and practitioners.

## Introduction

As a multinational country incorporating 56 officially recognized ethnic groups, China has long concerned itself with the stability and unity of its population's minority ethnic groups. Especially following the country's economic reform and introduction of policies opening it up to foreign trade and investment, which have had a profound influence on Chinese society overall, the Chinese government has promulgated a further series of policies that aim to promote interethnic harmony. Enhancing the mental health of the country's minority ethnic peoples is seen as an important part of this, yet, at the interchange between their own ethnic culture and that of the mainstream, this continues to be affected by several negative factors, including prejudice, discrimination, racialism, stereotyping, and stigmatization.

The first of these issues—“prejudice”—refers to a negative evaluation of a social group or an individual that is predominantly based on their group membership (Crandall and Eshleman, 2003). It is a significant risk factor for the mental health of members of minority ethnic groups, and can rapidly reverse the harmony of intergroup relations, with the latter even deteriorating into full-scale conflict, war, or genocide (Vasiljevic and Crisp, 2013). Moreover, prejudice does not simply disappear with the continuous improvement of social civilization. In fact, people who may have internalized unprejudiced values and norms can also hold genuinely prejudiced beliefs (Crandall and Eshleman, 2003). Additionally, members of the predominant group typically express prejudice intentionally (Plant and Devine, 2001, 2009) or under the veil of justification (Crandall and Eshleman, 2003), which leads them to seek out legitimizing myths to support the existing social arrangements (Jost and Banaji, 1994; Sidanius and Pratto, 1999). Meanwhile, members of minority groups perceive these prejudices every day (Don Operario and Fiske, 2001).

Although there are 55 minority ethnic groups in China, but the population of them is only 8.49% of the whole country. They inhabit a region or mix with Han ethnic, which is the biggest ethnic group of China. Generally, they have their own religion, costume and language. They experience communication and integration between local and other ethnic groups cultures. As a specific minority group in China, Chinese college students from minority ethnic groups also experience multicultural amalgamation in a distinct context, and thereby represent a source of hope for the advancement of ethnic minorities and a link to ethnic harmony in the future. Furthermore, relatively recently, the Chinese government advanced the idea of the “Chinese dream,” a set of individual as well as national ideals with which to accelerate the integration and rejuvenation of China. Playing an important role in the fulfillment of this ideology are the country's young people, including minority ethnic college students. Consequently, their mental health is of paramount importance. Yet, Chinese minority ethnic college students continue to experience prejudice every day, including the blind attribution of poor academic performance and life adaptation problems to their minority ethnic status, being required to follow a single standard of learning, and having to use Chinese instead of their own language. All such prejudices indicate that Chinese college students from minority ethnic groups are not easily accepted by members of the dominant ethnic group, primarily owing to the inherent differences between the dominant and minority ethnic groups.

As a society advances, prejudice may become gradually transformed and reappear in more subtle forms (Pettigrew and Meertens, 1995). These altered aspects may, in turn, facilitate a positive public image for an ostensibly revitalized culture, and be useful in the construction of a self-representation built in accordance with the principles of socially accepted tolerance (Salmeri and Pellerone, 2015). Correspondingly, it can be more difficult to measure these forms of prejudice. Therefore, the present study shifts its focus to “perceived prejudice.” Perceived prejudice has been defined as an individual's perception of an attitude, judgment, or evaluation not being consist with their actual situation, but instead related to the identity of the social group members (in this case, minority ethnic) with whom they are associated. Such perceptions can accurately correspond to the actual prejudice. Moreover, empirical research provides evidence that people who perceive prejudice from a dominant ethnic group experience stress and other unhealthy emotions such as anxiety, depression, terror, anger, hostility, and interpersonal sensitivity (Kim, 1988; van Zomeren et al., 2004; Barreto and Ellemers, 2005; Wang et al., 2012; Perry et al., 2015). In addition, for college students from minority ethnic groups, perceived prejudice not only causes stress and other unhealthy emotions, but also brings a negative influence to one's academic self-concept (Lehman, 2012). Hence, our research sets out to investigate perceived prejudice in relation to the mental health of Chinese ethnic minority college students.

Regarding the allied question of how perceived prejudice might negatively effect the mental health of Chinese ethnic minority college students, postmodern social constructionism theory suggests that a person is a construction of relationships and mentality is a construction of society (Yang, 2006). For Chinese college students from minority ethnic groups, perceived prejudice is a new relationship that can break the balance of previous relationships, and so these students need to reconstruct the new relationship to achieve new balance. In other words, their mentality will reconstruct after perceiving a prejudice, at least in the context of Chinese society. China is known for its collectivist culture, and, when members of minority ethnic groups perceive prejudice from the dominant ethnic group, they may identify their disadvantaged, weaker position and thus consider themselves not accepted by the mainstream. This, in turn, can bring about feelings of inferiority and otherwise not be beneficial for their mental health (Chen, 1997).

As well as the direct effects of perceived prejudice, there may also be some indirect effects to an individual's mental health. As noted, the Chinese government is increasingly focused on issues of ethnic identity, and has, among other meditations, begun assessing the likelihood of successful outcomes for Chinese minority ethnic college students following many years of educational practice. Further to these assessments, some new approaches have been implemented, including bilingual education and advanced-placement classes.

Previous studies have also identified ethnic identity to be an indispensable and incomparable “soft power” in the development of a nation under a specific social context. Assessments of the likelihood for success refer to an individual's positive psychological capital, which is very important to one's life development. If perceived prejudice indirectly brings about a negative reconstruction of Chinese minority college students' mental health through the two aspects, the harm to China as well as to the affected individuals is significant. Therefore, the present study undertakes to explore the influence of perceived prejudice on mental health from the perspectives of both the ethnic identity and the psychological capital of Chinese minority ethnic college students.

### Perceived prejudice, ethnic identity, and mental health

“Identity” is an evolving structure that incorporates individual identity and group identity. Individual identity is the identity of individual consciousness, or an individual's unconscious pursuit of the continuum of character. Group identity is the inner convergence of a group's ideals and characteristics. Each identity is a particular story, developed continuously, through perceptions, feelings, and thoughts, as well as through speeches, symbolic interactions, and memory (Salmeri and Pellerone, 2015). Ethnic identity is generally defined as an individual's perception of attribution and recognition of, and emotional attachment to, their own ethnic group (Zuo and Xiangrong, 2011), not only that, Phinney (2000) suggest the ethnic identity also includes the positive evaluation of their own ethnic group and participation in group activities. So in this study, we consider the changes to the development of the three aspects of ethnic identity that might occur when Chinese ethnic minority college students perceive prejudice, and what effect these could have on their mental health. In this regard, the rejection–identification model (Branscombe et al., 1999) suggests that minority group members perceiving rejection from an outgroup can reduce the effect of their mental health directly, enhancing their group identity and bringing about a positive influence on mental health at the same time. Following this theory, perceived prejudice can have positive indirect effects on mental health, and group identity is a mediating variable between perceived prejudice and mental health. Several empirical studies support this view (Schmitt et al., 2002; Garstka et al., 2004; Betts and Hinsz, 2013), but Chen (1997), while concurring that ethnic identity indeed acts as a mediator, found that it did not play a positive role for Chinese ethnic minority college students, seemingly supporting the idea of perceived prejudice leading to a negative reconstruction of ethnic identity in Chinese culture. The present study examines this apparent anomaly.

### Perceived prejudice, ethnic identity, hope, and mental health

Assessments of the possibility of success are considered in terms of “psychological capital,” comprising an individual's levels of confidence, optimism, resilience, and hope. Of these, “hope” is thought of as a state of positive motivation that is based on the interaction of the pathways to success and the agency in thinking to use the pathways (Snyder et al., 1991, 2000; Snyder, 2002). Within the Chinese government's initiatives to cultivate more motivation toward and offer support in planning pathways to success for Chinese minority ethnic college students, the hope aspect of psychological capital is clearly very important. But, how might perceived prejudice affect or even reconstruct hope? Symbolic interactionism theory suggests that an individual's self-concept is mainly constructed by evaluation based on feedback from important others (David and Thompson, 2005). When individuals perceive prejudice over the long term, they may internalize the prejudice as their own. This can affect their self-worth and gradually result in them manifesting behaviors consistent with negative stereotypes, as well as reduce the value of and motivation toward learning goals, which subsequently diminishes their levels of hope. This construal suggests that perceived prejudice would negatively influence hope. On the other hand, previous studies have found that hope positively influences the indices of mental health; for example, individuals with high levels of hope also have higher levels of life satisfaction and lower levels of hostility and suicidal ideation (Gilman et al., 2006; Marques et al., 2011), and hope has been shown to have a specific function that can alleviate psychological suffering (Berendes et al., 2010) and dysphoria levels (Kwon, 2000). In other words, hope may be a mediating variable between perceived prejudice and mental health.

**Figure 1:**
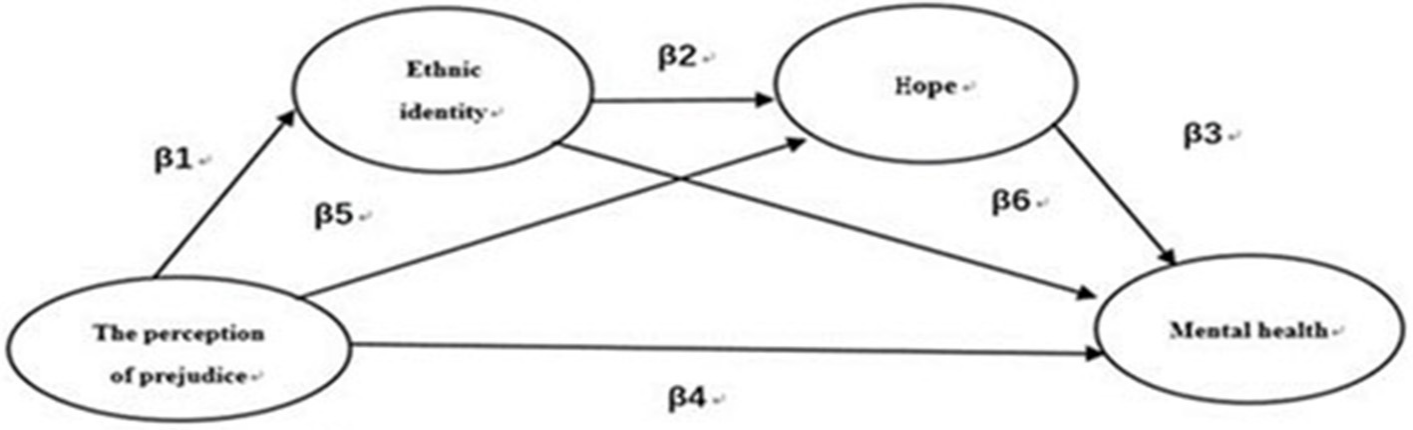
The hypothesis model of the relationship between perceived pre judice and mental health.

Community psychology, in explaining the relationship between ethnic identity and hope, suggests that communities were formed for the common benefit, offering affiliation and meeting the needs and emotional connection of their members. Members of a community are seen to commit to the common benefits of the group and gradually form a unified behavior that is used to achieve common benefit goals. These goals are based on a sense of affiliation and of dependence concerning the communities. Chinese ethnic minority college students, although they may come from many different minority ethnic groups, can nonetheless form a community with common benefit goals. In this kind of community, too, ethnic identity can provide a sense of affiliation and dependence, and may lead to the production of goals, pathways, and agency. In other words, ethnic identity may have positive effects with respect to hope in our study's context. Thus, the full hypothesis for this paper is that, while perceived prejudice may negatively influence mental health, this relationship was mediated by ethnic identity and hope in Chinese college students from minority ethnic groups. The study's mediation model is shown in Figure 1, which illustrates three mediation paths: β1–β6, β5–β3, and β1–β2–β3 (Taylor et al., 2008).

## Materials and methods

### Participants and procedure

We choose nine colleges from the Guangxi Zhuang autonomous region and Yunnan and Guizhou provinces, which encompass many Chinese minority ethnic groups. The type and grade of each college is listed in Table 1.

**Table 1:** Type and grade of schools attended by the study's participants^a^.

**School name**	**Type**	**Grade**	***n***	**%**
Guizhou Normal University	Higher education	Provincial key university	53	8
Guizhou University of Finance and Economics	Higher education	Provincial key university	60	9
Guizhou Minzu University	Higher education	Provincial–Ministry co-constructed university	99	15
Guiyang College of Traditional Chinese Medicine	Higher education	Provincial key university	53	8
Guizhou Medical University	Higher education	Provincial key university	47	7
Wenshan University	Higher education	Ordinary university	53	8
Guangxi Electrical Polytechnic Institute	Post-secondary vocational training	Ordinary vocational college	153	23
Nanning College for Vocational Technology	Post-secondary vocational training	Ordinary vocational college	80	12
Guangxi Normal University	Higher education	Provincial–Ministry co-constructed university	67	10

a *N = 665*.

**Figure 2:**
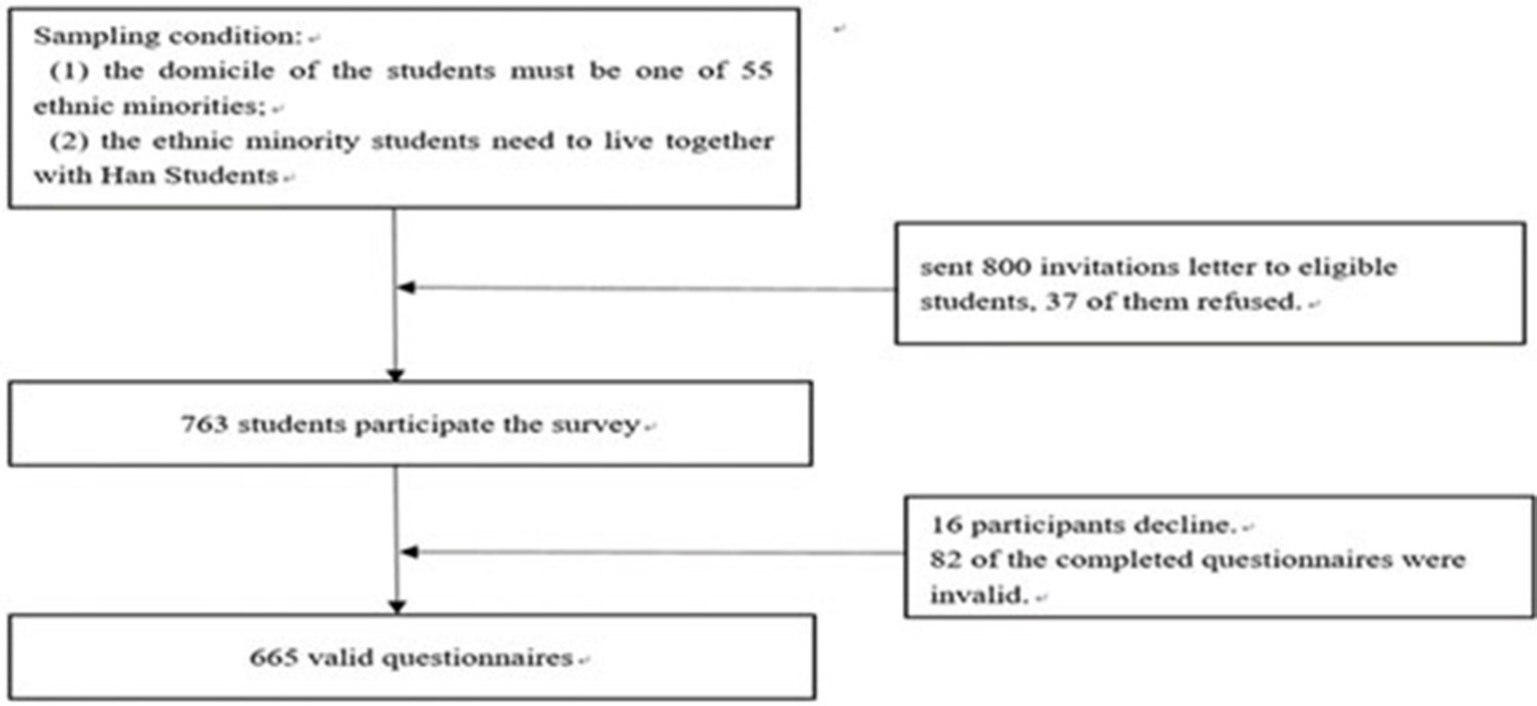
Flow diagram of the selection process used in this study. The search was conducted on 15 December 2015.

To be selected to participate in the study, students had to, first, be a member of one of China's 55 officially recognized minority ethnic groups, and, second, be living alongside Han Chinese students (Han Chinese being the country's largest ethnic group). We sent 800 invitation letters to eligible participants in December 2015; 37 of them declined to participate in the survey, resulting in a response rate of 95.38%. In the process of completing the questionnaire, 16 participants withdrew and 82 of the completed questionnaires were invalid; 665 remaining questionnaires were valid (87.16%). Figure 2 charts the complete selection process.

The age of participants ranged from 18 to 26 years old (see Table 2 for detailed demographic data), and the information of the participants' ethnicity are showed in Table 3. No payment was issued for involvement in the study, and the questionnaires were completed in a classroom environment. Prior to their completing the questionnaires, verbal informed consent was obtained from all participants. They did not include their names on the questionnaires, and were assured of the confidentiality of their responses. The questionnaires took approximately 30 min to complete.

**Table 2:** Demographics of the study's participants^a^.

**Demographic variables**		***n***	**%**	**Demographic variables**		***n***	**%**
Ethnicity	Mongols	4	0.6	Gender	Male	207	31.3
	Hui	16	2.41		Female	458	68.7
	Tibetan	3	0.45	Family incomes (RMB, ¥)	<20,000	453	68.1
	Hmong	119	17.89		20,000–50,000	127	19.1
	Yi	58	8.85		50,000–80,000	35	5.26
	Bourau	189	38.42		>80,000	22	3.31
	Buxqyaix	59	8.87		Missing	28	4.21
	Manchu	1	0.15	Mother's education level	Illiteracy	128	19.3
	Gaeml	42	6.32		Primary school or Junior High School	410	61.7
	Yao	17	2.56		High school or Technical Secondary School	69	10.4
	Bai	35	5.26		University, college or above	45	6.77
	Tujia	44	6.62		Missing	13	1.95
	Hani	10	1.5	Type of School	Ethnic school	127	19.1
	Dai	4	0.6		Non-ethnic School	513	77.1
	Li	2	0.3		Missing	25	3.76
	Lisu	2	0.3	Grade	Freshman	227	34.1
	Wa	3	0.45		Sophomore	257	38.7
	Shui	8	1.2		Junior and Senior	175	26.3
	Naxi	10	1.5		Missing	6	0.9
	Tu	1	0.15	Religion	Non-religious	601	90.4
	Chiang	3	0.45		Christianity	2	0.3
	Gelo	15	2.26		Mohammedanism	14	2.11
	Achang	1	0.15		Buddhism	43	6.46
	Pumi	2	0.3		Others	4	0.6
	others	16	2.41		Missing	1	0.15
	Missing	1	0.15	Father's education level	Illiteracy	24	3.61
Districts	Countryside	490	73.68		Primary school or Junior High School	424	63.8
	Town	127	19.1		High school or Technical Secondary School	136	20.5
	City	37	5.56		University, college or above	67	10.1
	Missing	11	1.66		Missing	14	2.11

a *N = 665*.

**Table 3:** Information of the participants' ethnicity.

**Ethnicity**	**General population (million)**	**Language**	**Religion**
Mongols	6.50	Mongolian	Mongolian, Shamanism, Tibetan Buddhism, Mohammedanism
Hui	9.81	Chinese, Jingtang language	Mohammedanism
Tibetan	7.50	Tibetan language	Tibetan Buddhism, Bonism
Hmong	9.43	Hmong language	Nature and ancestor worship
Yi	8.71	Yi language	Nature and ancestor worship
Bourau	15.00	Zhuang language	MOZ, Nature, and ancestor worship
Buxqyaix	2.87	Bouyei language	MOZ, Catholic Church, Polytheistic worship
Manchu	10.41	Manchurian, Chinese	Shamanism, Buddhism, Most of people have no faith
Gaeml	2.87	Dong Language	Polytheism
Yao	2.85	Yao language	Nature and ancestor worship, totemism, Shamanism, Taoism
Bai	1.93	Bai language	Local deity worship, Taoism, Christianity
Tujia	8.35	Tujia Language	Nature, ancestor and Hero worship, Totemism, Taoism
Hani	1.63	Hani language	Polytheism, Ancestor worship
Dai	1.23	Dai language	Theravada Buddhism, Primitive religion, Hinduism
Li	1.49	Li language	Nature and ancestor worship, Totemism
Lisu	0.80	Lisu language	Primitive religion, Christianity
Wa	0.43	Wa language	Primitive religion, Buddhism, Christianity
Shui	0.41	Sui language	Polytheism
Naxi	0.31	Naxi language	Dongbaism
Tu	0.28	Monguor language	Lamaism, Taoism, Polytheism, Shamanism
Chiang	0.31	Qiang language	Primitive religion, Nature worship
Gelo	0.58	Gelao language, Chinese	Ancestor worship, Polytheism, Taoism, Buddhism
Achang	0.07	Achang language	Theravada Buddhism, Animism, Ancestor worship
Pumi	0.08	Primi language	Bonism, Han gui teach, Tibetan Buddhism

*MOZ is a special religion of Bourau and Buxqyaix, which integrates Buddhism and Taoism*.

### Ethics statement

Review and approval processes were not required for this study, in accordance with institutional and national requirements.

### Measures

#### Perceived prejudice

To make the perceived prejudice scale more suitable for use by Chinese college students from minority ethnic groups, we translated and revised a related scale developed by Stephan et al. (1998). The adapted scale included 12 items and two dimensions: perceived positive prejudice and perceived negative prejudice, Sample items included: “I felt excluded from the Han group because of my ethnicity” and “I feel that Han individuals are prejudiced regarding my ethnic group.” Each item was scored on a 5-point scale, ranging from 1 (*strongly disagree*) to 5 (*strongly agree*). Confirmatory factor analysis showed that the fit indexes for χ^2^/*df* = 5.09, Tucker–Lewis index (TLI) = 0.93, comparative fit index (CFI) = 0.95, and root mean square error of approximation (RMSEA) = 0.078. The indicators of the model fit were accepted. The Cronbach alpha coefficient for the perceived prejudice scale was 0.82.

#### Ethnic identity

The study used a revised version of the Multigroup Ethnic Identity Measure (Phinney, 1992) and the Ethnic Identity Scale (Unmaña-Taylor et al., 2004) utilized by Gao et al. (2011). The scale included 15 items and three dimensions: ethnic exploration, ethnic affirmation and ethnic confirmation. Sample items included “As a member of my ethnic group, I feel very happy” and “I have a strong attachment to my own ethnic group.” Each item was scored on a 4-point scale, ranging from 1 (*strongly disagree*) to 4 (*strongly agree*). The Cronbach alpha coefficient for the ethnic identity scale was 0.88.

#### Hope

Hope was measured by using the Adult Dispositional Hope Scale originated by Snyder et al. (1991) and revised by Chen et al. (2009). The scale included two dimensions—covering feelings of agency and pathways toward goals—and each dimension had four items. Sample items included “I always tirelessly pursue my goals” and “For any problems, there will be many solutions and measures.” While the scale comprised two dimension and four interference items, the interference items were not included in the total score. The evaluation was based on a 4-point scale, from 1 (*strongly disagree*) to 4 (*strongly agree*). The Cronbach alpha coefficient for the hope scale was 0.78.

#### Mental health

Mental health was measured by using the General Health Questionnaire revised by Li and Boey (2002). The scale included 20 items and three dimensions: self-affirmation, anxiety, and depression. Sample items included “Are you satisfied with your way of solving problems?” “Are you feeling unhappy or moody?” and “Are you feeling like a useless person?” The scale was scored based on “yes” (1 point) or “no” (0 points) answers. The dimensions of anxiety and depression required reverse scoring. Higher scores indicated a higher level of mental health. Confirmatory factor analysis showed that the fit indexes for χ^2^/*df* = 1.79, IFI = 0.95, CFI = 0.94, and RMSEA = 0.039. The Cronbach alpha coefficient for the mental health scale was 0.77.

### Data analyses

#### Common method bias control

Because all of the data were generated by self-report, they could have been affected by common method bias, which might, in turn, decrease the validity of the results. Approaches for controlling for common method bias include “process control” and “statistical control.” Process control refers to control measures incorporated into the process of a study's design and measurement by researchers. The questionnaires of our study were only used for academic research, and not for the use of any organization or individuals. Participants' information was kept strictly confidential. Based on the principles of voluntary participation, the study's respondents could suspend their involvement with the questionnaire(s) at any time. Moreover, they were asked to select options that most accorded with the actual situation—there were no “right” or “wrong” answers. We used the class collective measured approach to acquire the data, and recycled the questionnaires immediately after each survey was completed. Each of these methods has been shown to control common method bias.

In addition, statistical control involves a statistical test that is applied after data collection—we usually used the Harman single factor test to test for common method bias. The results showed that 18 factors had an eigenvalue greater than 1, and the first factor accounted for 20.85% variance, which is less than the 40% of the critical standard. This also shows that common method bias was not apparent.

Finally, in terms of data analysis, all data in this study were analyzed using SPSS version 20.0 (IBM) and Mplus version 7.0 (Muthén and Muthén).

#### Structural equation modeling (SEM)

The structural equation modeling process comprised four steps (Li, 2011): (1) data feature checking (data should have a multivariate normal distribution, and hence no serious collinearity problems); (2) ensuring the measurement model of confirmatory factor analysis results conforms to the requirements; (3) construction of the structural equation model and model fitting analysis; (4) model modification.

#### Multiple mediation effects test

There are various methods with which to test for multiple mediation effects, with the two most common tests being “joint significance” and the “bootstrap test” (Taylor et al., 2008). A test of joint significance determines whether the mediation effect of each path coefficient is significant, and, if so, whether the mediation effect is significant. This method is relatively simple, but it cannot estimate the confidence interval of the mediation effect, and the condition demands are too strict. Conversely, the bootstrap test can estimate the confidence interval of the mediation effect, and so is a comparatively advantageous test method. Accordingly, the present study used the bootstrap test method.

## Results

### Descriptive statistics

The mean value, standard deviation, and correlation coefficients of each variable were calculated, and the results are presented in Table 4. Perceived prejudice was significantly negatively correlated with ethnic identity, hope, and mental health. Ethnic identity, hope, and mental health were significantly positively correlated with each other. This explains, to some extent, how perceived prejudice influences ethnic identity, hope, and mental health. In addition, it also shows the effects of ethnic identity on hope and mental health, and the meaning of hope for mental health.

**Table 4:** Means, standard deviations, theoretical minimums and maximums, and intercorrelations.

		**1**	**2**	**3**	**4**
1	The perception of prejudice	1			
2	Ethnic identity	−0.293^***^	1		
3	Hope	−0.291^***^	0.425^***^	1	
4	Mental health	−0.288^***^	0.233^***^	0.616^***^	1
	*M*	26.74	46.29	23.01	14.32
	*SD*	6.58	7.22	3.42	3.58
	*Theoretical Min*.	12	15	8	0
	*Theoretical Max*.	60	60	32	20

*N = 665*.

*** *p < 0.001, all tests were two-tailed*.

The predictive power of the demographic variables on perceived prejudice and ethnic identity is demonstrated in Tables 5, 6. The data indicate that all demographic variables had no significant effect on perceived prejudice (*F* = 1.137, *p* > 0.05) and ethnic identity (*F* = 1.299, *p* > 0.05). Thus, it was inferred that demographic variables did not have a significant effect on the construction of the chain mediation model.

**Table 5:** Model of multiple linear regression analyses predicting perceived prejudice.

**Model**		**Sum of Squares**	***df***	**Mean square**	***F***	***R^2^***	***Adjust R^2^***	***SE***
1	Regression	47.240	42	1.125	1.137	0.079	0.010	0.994
	Residual	551.787	558	0.989				
	Total	599.027	600					

*Demographic variables included age, gender, type of school, ethnicity, family income, religion, and levels of education of father and mother. Multiple linear regression analyses after transforming the categorical variables into dummy variables*.

**Table 6:** Model of multiple linear regression analyses predicting ethnic identity.

**Model**		**Sum of Squares**	***df***	**Mean square**	***F***	***R^2^***	**Adjust *R^2^***	***SE***
1	Regression	53.394	42	1.271	1.299	0.089	0.020	0.989
	Residual	546.171	558	0.979				
	Total	599.564	600					

*Demographic variables include age, gender, type of school, ethnicity, family income, religion, and levels of education of father and mother. Multiple linear regression analyses after transforming the categorical variables into dummy variables*.

### Construction of chain mediation model

**Figure 3:**
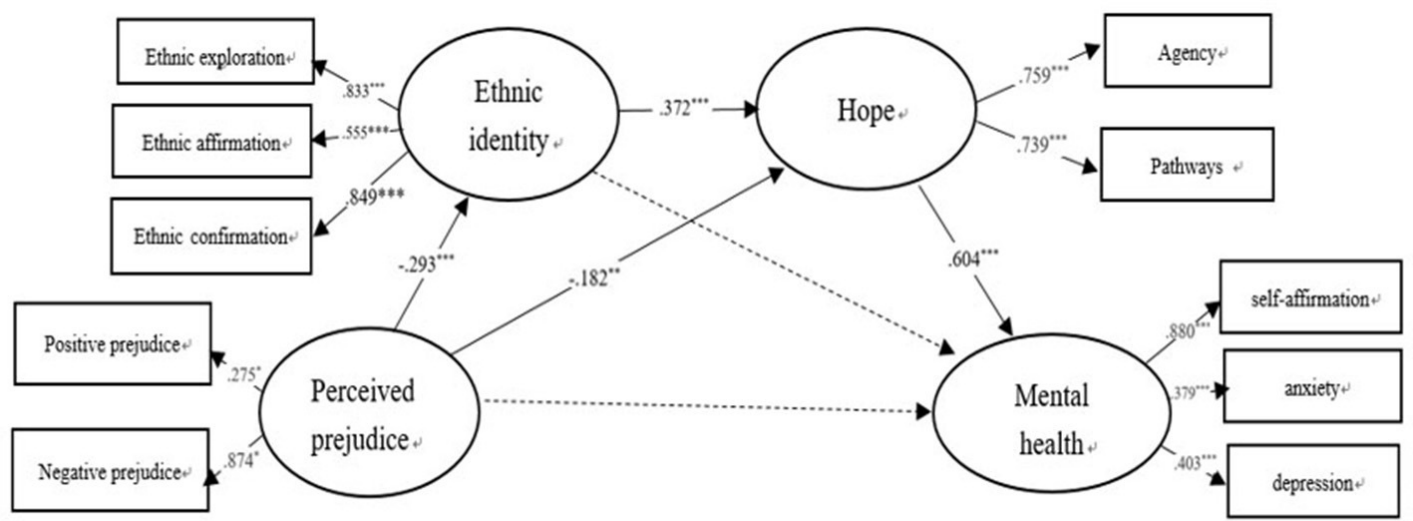
The multiple mediator model of the perception of prejudice and mental health. (The dotted line is not significant path). Perceived prejudice, Ethnic identity, Hope and Mental health are latent variables, and have some different indicators. Each indicator is the sum scores of dimensions of latent variables. ^*^p < 0.05, ^**^p < 0.01, ^***^p < 0.001.

We tested whether the data were consistent with normal distribution by measuring the skew and kurtosis distributions. The absolute value of the coefficient of skew (*Z*s) and kurtosis (*Z*k) was less than 1.96, which shows that the data conform to the multivariate normal distribution. Collinearity diagnostics showed tolerance (0.946, 0.883, and 0.887) to be greater than 0.10, and the variance inflation factor (1.057, 1.133, and 1.414) was less than 10. Therefore, no serious collinearity problem was indicated. Confirmatory factor analysis showed that the measurement model fit the data well [RMSEA = 0.065, CFI = 0.942, TLI = 0.907, and the standardized root mean square residual (SRMR) = 0.043]. The test of the hypothesized multiple mediator model, as illustrated in Figure 3, resulted in a good fit to the data (RMSEA = 0.071, CFI = 0.940, TLI = 0.903, and SRMR = 0.043).

### The mediating effect test

We used the deviation correction nonparametric percentage test of bootstrap, repeated sampling 1,000 times, and calculated 95% confidence intervals (CI). The finalized structural model (see Figure 3) shows that the path from perceived prejudice to mental health through ethnic identity was non-significant; however, the other paths from perceived prejudice to mental health through hope and the chain of ethnic identity and hope were significant. Confidence intervals were 95% CI [−0.210, −0.010] and [−0.104, −0.027]. The value of the total mediating effect was −0.158, *p* < 0.01, and the 95% CI [−0.259, −0.056] did not overlap with zero, indicating that the total mediating effect was significant; the size of effect was 54.9%. The value of specific indirect effects from perceived prejudice to mental health through hope and the chain of ethnic identity and hope were −0.110, *p* < 0.05 and −0.066, *p* < 0.01, and the size of effects were 38.2 and 22.9% respectively.

### The multi-group analysis of SEM

We used multi-group analysis to analyze whether the multiple mediator model differ significantly across all ethnic groups (Conditions for comparison: *n* > 20). So four models are set up to compare. The first model has no constraint to the model parameters; the second model constrains the measurement weights to be equal; the third model constrains the measurement weight and structural weight to be equal; the fourth model constrains the measurement weight, structural weight, structural covariances, structural residuals and measurement residuals to be equal. The following fit indices were generated by a SEM analysis. The result shows non-significant Chi-square differences among the four models, Δχ^2^ = 74.54, *P* > 0.05, as well as the slightly smaller AIC value indicated that the multiple mediator model was not found significant difference across ethnicity, lending preliminary support to its robustness.

## Discussion

The present study investigated the influence of perceived prejudice on mental health through ethnic identity and hope in Chinese college students from minority ethnic groups. Our results suggest that perceived prejudice can negatively influence mental health and bring about negative reconstruction to ethnic identity and hope within the context of Chinese culture. Ethnic identity and hope act as mediators in the relationship between perceived prejudice and mental health. The size of the total mediating effect was 54.9%, which was greater than the size of the direct effect (45.1%). This indicates that the constructed model has significant explanatory power to describe the negative influence of perceived prejudice on mental health. The model features two significant paths (see Figure 1), the first of which is from perceived prejudice to mental health through hope (β5–β3), indicating that perceived prejudice has a significant, negative reconstructive effect on hope. Hope is an important psychological resource that has substantial meaning for constructed values and the achievement of success. When the Chinese ethnic minority college students perceived greater amounts of prejudice, their motivation for achieving goals lessened, compared to that of students from the dominant ethnic group, in turn reducing their levels of hope and generating feelings of worthlessness and meaninglessness, resulting in reduced self-affirmation, increased anxiety, and depression, and hence ultimately affecting their mental health. The second path observed in the constructed model is that from perceived prejudice to mental health through the chain of ethnic identity and hope (β1–β2–β3). This signified that the effect of perceived prejudice through ethnic identity alone did not produce a significant influence on mental health—it needed to act on hope as well. Meanwhile, we found that ethnic identity partially mediated the relationship between perceived prejudice and hope. In addition, the results show that perceived prejudice negatively predicted ethnic identity, which is inconsistent with the rejection–identification model but supports Chen's (1997) standpoint. This suggests that perceived prejudice prompts Chinese ethnic minority college students to negatively reconstruct their ethnic identity in the context of Chinese culture.

## Conclusion

### Directions for future research

The present research has important implications for researchers and practitioners alike. For researchers, this study offers some indication, at least, that other kinds of group identity (e.g., national identity) and other components of psychological capital (e.g., self-efficacy) may also play an important role in the relationship between perceived prejudice and mental health. For practitioners, it gives some suggestions toward reducing the negative influence of perceived prejudice to mental health in Chinese ethnic minority college students. For example, it is important to make efforts to prevent perceived prejudice becoming a significant component of the reconstruction of negative “meaning” to ethnic identity and hope. In addition, ethnic identity and hope should be enhanced over other mechanisms to compensate for losses brought about by perceived prejudice.

### Limitations

A few limitations of the present study also merit consideration. First, the results were based on self-reports, and this approach has potential problems, including that a participant's responses may not be sufficiently accurate or that the responses may also have been subject to the social desirability effect. Second, the fact that this study essentially corresponds to related research does not prove causality. Follow-up research should design experiments or longitudinal studies to supplement and validate our results, and potentially improve the external validity of the study. Third, the lack of balancing of some demographic variables in our sample may limit the application of the results in different groups.

In conclusion, the present study provides some insight into the relationship between perceived prejudice and mental health. Specifically, we found that perceived prejudice negatively influences mental health through ethnic identity and hope in Chinese ethnic minority college students. The relationship between perceived prejudice and mental health was fully mediated by hope and the chain of ethnic identity and hope. Ethnic identity partially mediated perceived prejudice and hope. As can be inferred from the size of the mediating effect, ethnic identity and hope play an important role in perceived prejudice and mental health, and, in our analysis, perceived prejudice was shown to prompt Chinese ethnic minority students to negatively reconstruct their ethnic identity and hope mechanisms, at least in the context of contemporary Chinese culture.

## Author contributions

Individual contributions of the named authors are as followed: JY has participated in the design, data collection, data analysis, data interpretation and drafting the early version of the article. LY has participated in the design, data collection and revising the article critically for better intrinsic logicality. The total number of words in the article is 5202.

### Conflict of interest statement

The authors declare that the research was conducted in the absence of any commercial or financial relationships that could be construed as a potential conflict of interest. The reviewer EP and handling Editor declared their shared affiliation, and the handling Editor states that the process nevertheless met the standards of a fair and objective review.
